# Doinseunggitang Ameliorates Endothelial Dysfunction in Diabetic Atherosclerosis

**DOI:** 10.1155/2013/783576

**Published:** 2013-08-25

**Authors:** Jung Joo Yoon, Yun Jung Lee, Ok Ju Park, So Min Lee, Yong Pyo Lee, Nam Geun Cho, Dae Gill Kang, Ho Sub Lee

**Affiliations:** ^1^College of Oriental Medicine and Professional Graduate School of Oriental Medicine, Wonkwang University, Shinyong-dong, Iksan 570-749, Jeonbuk, Republic of Korea; ^2^Hanbang Body-fluid Research Center, Wonkwang University, Shinyong-dong, Iksan 570-749, Jeonbuk, Republic of Korea; ^3^Department of Acupuncture & Moxibustion Medicine, Oriental Medicine hospital, Wonkwang Universitiy, Republic of Korea

## Abstract

Atherosclerosis, a chronic and progressive disease characterized by vascular inflammation, is a leading cause of death in diabetes patients. Doinseunggitang (DYSGT), traditional prescription, has been used for promoting blood circulation to remove blood stasis. The aim of this study was to investigate the beneficial effects of DYSGT on endothelial dysfunction in diabetic atherosclerosis animal model. Apolipoprotein E knockout (ApoE KO) mice fed on a Western diet were treated with DYSGT (200 mg/kg/day). DYSGT significantly lowered blood glucose level and glucose tolerance as well as systolic blood pressure. Metabolic parameter showed that DYSGT markedly decreased triglyceride and LDL-cholesterol levels. In the thoracic aorta, the impairment of vasorelaxation response to acetylcholine and atherosclerotic lesion was attenuated by DYSGT. Furthermore, DYSGT restored the reduction of endothelial nitric oxide synthase (eNOS) expression, leading to the inhibition of intracellular adhesion molecule-1 (ICAM-1) and endothelin-1 (ET-1) expression. In conclusion, DYSGT improved the development of diabetic atherosclerosis via attenuation of the endothelial dysfunction, possibly by inhibiting ET-1, cell adhesion molecules, and lesion formation. Therefore, these results suggest that Korean traditional prescription Doinseunggitang may be useful in the treatment and prevention of diabetic vascular complications.

## 1. Introduction

Atherosclerosis and the associated cardiovascular disease (e.g., myocardial infarction, stroke, and peripheral vascular disease) are the principal cause of morbidity and mortality in diabetes patients. Atherosclerosis is defined as a chronic and progressive disease characterized by an inflammatory response of the arterial wall [1–3]. Vascular tone is an important factor in regulation of arterial blood pressure. Changes in vascular smooth muscle tone and the internal diameter of vessels can profoundly alter tissue perfusion and can impair the ability of arteries to respond to vasodilators and vasoconstrictors [4, 5]. The endothelium-dependent vasorelaxation that is induced by acetylcholine (ACh) is mediated by nitric oxide (NO), which acts through soluble guanylyl cyclase and cyclic GMP. Thus, this phenotypic change appears to result from a decline in NO bioavailability due to impaired NO biosynthesis and inactivation of NO by superoxide, which leads to hypertension. These impaired vascular responses are also shown in hypercholesterolemia and obesity [6, 7]. 

Endothelin (ET-) 1 expression is significantly higher in aortic and mesenteric arteries of hypertensive animal models. Hypertensive patients with high plasma ET-1 levels often exhibit elevated cell adhesion molecule levels and increased risks for developing hypertension-induced organ damage [8]. One early phase of atherosclerosis involves the recruitment of inflammatory cells from the circulation and their endothelial migration [9]. This process is predominantly mediated by cellular adhesion molecules, which are expressed on the vascular endothelium and on circulating leukocytes in response to several inflammatory stimuli. Selectins and their ligands are involved in the rolling and tethering of leukocytes on the vascular wall. Intracellular adhesion molecule-1 (ICAM-1) induces firm adhesion of inflammatory cells at the vascular surface [10].

Peroxisome proliferator activated receptors (PPARs) are a family of nuclear transcription factors, of which there are four members: *α*, *β*, *γ*-1, and *γ*-2. PPAR-*γ* plays an important role in regulating inflammatory, and PPAR-*γ* agonist has been shown to reduce atherosclerosis in hypercholesterolemia or hyperplasia [11, 12]. It is highly expressed in all major cell types participating in atherosclerotic process to regulate transcription of a variety of genes encoding proteins involved in glucose and lipid metabolism. 

Although atherosclerosis is not a distinguishing feature described in ApoE-deficient humans [13], ApoE-deficiency alone proved to be sufficient for aortic atherosclerotic plaques to develop in mice. In addition, high fat and cholesterol diet markedly accelerate plaque development in these mice [14]. The lesion development and plaque composition in ApoE KO mice are also similar to those in humans, establishing them as an excellent animal model for studying the pathogenesis of atherosclerosis. 

Doinseunggitang (Taohe Chengqi Tang: Chinese) which is a traditional medicinal prescription has been used orally for promoting blood circulation to remove blood stasis. Doinseunggitang is one of the herbal mixtures documented in Shang Han Lun and this prescription is composed of *Glycyrrhizae uralensis *Fischer, *Rheum undulatum* Linne, *Prunus persica* L., and *Cinnamomum cassia *Presl. In clinic, Doinseunggitang has been documented to treat chronic hepatitis, amenorrhea, diabetes mellitus, acute necrotic enteritis, and chronic pyelonephritis [15]. However, the action mechanisms for this effectiveness of Doinseunggitang remained obscure. Here, we investigated the beneficial effects of Doinseunggitang (DYSGT) on vascular dysfunction in Western-diet-fed ApoE KO mice.

## 2. Methods

### 2.1. Preparation of DYSGT

The formula of DYSGT consists of five herbs including *Glycyrrhizae uralensis *Fischer (15 g), *Rheum undulatum* Linne (75 g), *Prunus persica* L. (37.5 g), and *Cinnamon cassia *Presl. (7.5 g) mixed and ground into a crude powder. The DYSGT (135 g) was boiled with 1 L of distilled water at 100°C for 2 h. The extract was centrifuged at 990 ×g for 30 min at 4°C and resulting supernatant was lyophilized to produce a powder (12.45 g), which was then kept at −70°C until using this experiment.

### 2.2. Experimental Animals

Six-week-old male ApoE gene deficient C57BL6J mice (ApoE KO) and normal C57BL6J mice were obtained from Central Lab. Animal Inc. (Seoul, Republic of Korea) and housed in metabolic cages with an automatic temperature and relative humidity (22 ± 2°C, 50~60%), and lighting (12 h light/dark cycle) condition. They were given free access to food and DW, and the consumptions were measured biweekly, respectively. They were fed a pelletized commercial chow diet for acclimatization for 2 weeks on arrival. After acclimatization, animals were randomly divided into four groups (*n* = 12), namely, (1) the control group (C57BL6J mice + regular diet + DW), (2) ApoE KO control group (ApoE KO + Western diet + DW), (3) positive control group (ApoE KO + Western diet + rosiglitazone 10 mg/kg/day), (4) DYSGT group (ApoE KO + Western diet + DYSGT 200 mg/kg/day). The peroxisome proliferator-activated receptor-*γ* (PPAR-*γ*) agonist, rosiglitazone, was chosen as a positive control, which is an antidiabetic agent for the treatment of type 2 diabetes. The control and ApoE KO control groups received regular diet and Western diet, respectively, for 12 weeks. The Western diet (WTD) was purchased from Research Diets, Inc. (Table 1). 

### 2.3. Measurement of Blood Pressure (SBP)

SBP was determined using a noninvasive tail-cuff monitor (MK2000; Muromachi Kikai, Tokyo, Japan). At least eight determinations were made in every session, and the mean of the lowest five values within 5 mmHg was taken as the SBP level. 

### 2.4. Plasma Biochemical Analysis

The concentration of glucose in blood was measured with whole blood sample obtained from vein of tail using a One Touch Ultra Blood Glucose Meter and Test Strip (Life Scan Inc., CA) at biweekly, respectively. Blood samples were taken by periorbital vein for biochemical analysis. Plasma insulin levels were measured based on ELISA method using commercial mice insulin ELISA kit (Shibayagi Co., Gunma, Japan). LDL cholesterol, total protein, triglyceride (TG), blood urea nitrogen (BUN) levels in plasma were enzymatically measured using a commercially available kits (ARKRAY, Inc., Minami-ku, Kyoto, Japan).

### 2.5. Quantitative Histopathology

Aortae isolated from all groups were fixed in 10% (v/v) formalin in 50 mM potassium phosphate buffer (pH 7.0) for 48 h at 4C. The tissues were subsequently embedded in paraffin and cross-sections (6 *μ*m) of the aortic arch in each group were stained by use of hematoxylin and eosin (H&E). For quantitative histopathologic comparisons, the mean of 10 sections was taken and intima-to-media ratio was determined by Axiovision 4 Imaging/Archiving software (Axiovision 4, Carl Zeiss, Germany).

### 2.6. Measurement of Atherosclerotic Lesions by Oil Red O Staining

 Mice were euthanized, and thoracic and abdominal aorta were used for en face staining with Oil Red O to visualize neutral lipid (cholesteryl ester and triglycerides) accumulation. In brief, the aorta was removed, cleaned, and cut open with the luminal surface facing up and then immersion-fixed in 10% formalin in 10 mM phosphate-buffered saline. After rinsing with phosphate-buffered saline, the aorta was thoroughly cleaned of adventitial fat using microforceps and spring iris scissors under a stereoscopic microscope. The inner aortic surface was stained with Oil Red O for 25 min at room temperature after rinsing with 60% isopropyl alcohol and distilled water. Images of Oil red O stained aortas were taken with a Axiovision 4 Imaging/Archiving software (Axiovision 4, Carl Zeiss, Germany).

### 2.7. Recording of Isometric Vascular Tone

Vascular tone was determined as previously described by Kang et al. [16]. At the end of the experiment, mice were sacrificed by decapitation. The thoracic aorta was rapidly and carefully dissected and placed into ice-cold Krebs solution (118 mM NaCl, 4.7 mM KCl, 1.1 mM MgSO_4_, 1.2 mM KH_2_PO_4_, 1.5 mM CaCl_2_, 25 mM NaHCO_3_, and 10 mM glucose, pH 7.4). The aortae were removed free of connective tissue and fat and cut into rings with a width of approximately 3 mm. All dissecting procedures were done with extreme care to protect the endothelium from inadvertent damage. The aortic rings were suspended by means of two L-shaped stainless steel wires inserted into the lumen in a tissue bath containing Krebs solution at 37°C. A gas mixture of 95% O_2_ and 5% CO_2_ was continuously bubbled through the bath. The base line load placed on the aortic rings was 1.0 g. Changes in isometric tension were recorded using a Grass model FT 03 force displacement transducer (Grass Technologies, Quincy, MA) connected to a model 7E polygraph recording system (Grass Technologies). The aortic relaxation by the cumulative addition of ACh was performed in the presence of endothelium.

### 2.8. Immunohistochemical Staining of ET-1, ICAM-1, and eNOS in Aortic Tissue

Slides were immunostained by Invitrogen's Histostain-SP kits using the Labeled-Strept-avidin-Biotin (LAB-SA) method. Slides were immersed in 3% hydrogen peroxide for 1 min at room temperature to block endogenous peroxidase activity and rinsed with PBS. And then, slides were incubated with 10% nonimmune goat serum for 20 min at room temperature to block nonspecific staining and incubated with a primary antibodies of ET-1, ICAM-1, and eNOS (Santa Cruz Biotechnology, Santa Cruz, CA) at a final dilution of 1 : 1000, in humidified chambers for overnight at 4°C. All slides were incubated with biotiny-lated secondary antibody for 20 min at room temperature and then incubated with horseradish-peroxidase-conjugated streptavidin for 20 min at room temperature, followed by detection with 3-amino-9-ethylcarbazole (AEC) as chromogen and counterstaining with hematoxylin (Zymed, CA). For the quantitative analysis, the average score of 10–20 randomly selected area was calculated using NIH Image analysis software, Image J (NIH, Bethesda, MD).

### 2.9. Immunofluorescence Staining of ICAM-1 and ET-1 in Aortic Tissue

Slides of the pancreatic frozen section were incubated with 10% nonimmune goat serum for 1 h at room temperature to block nonspecific staining, and incubated with primary antibodies of ICAM-1, and ET-1 (1 : 100, Santa Cruz Biotechnology) for overnight at 4°C. After washing, fluorescein-conjugated goat anti-rabbit IgG and Alexa Fluor 594-conjugated donkey anti-goat IgG (1 : 200, Molecular Probes, Carlsbad, CA) were incubated for 1 h at room temperature. After washing 3 times, the sections were mounted and observed by Olympus fluorescence microscopy. The expressions of insulin in aortic tissue were observed by Olympus microscopy equipped with an Olympus DP 70 camera. For the quantitative analysis, the average score of 10–20 randomly selected area was calculated using NIH Image analysis software, Image J (NIH, Bethesda, MD).

### 2.10. Protein Preparation and Western Blot Analysis

Thoracic aortae and muscle were homogenized in a buffer consisting of 250 mM sucrose, 1 mM EDTA, 0.1 mM phenylmethylsulfonyl fluoride, and 20 mM potassium phosphate buffer (pH 7.6). Large tissue debris and nuclear fragments were removed by two successive low-speed spins (3,500 rpm, 5 min; 8,000 rpm, 10 min, 4°C). The recovered protein (40 *μ*g) was separated by 10% SDS-PAGE and transferred electrophoretically to nitrocellulose membranes using a Mini-Protean II apparatus (Bio-Rad, Hercules, CA). Membranes were blocked with 5% nonfat milk powder in 0.05% Tween 20-phosphate buffered saline (PBS-T) for 1 h prior to incubation in the presence of primary antibodies to ET-1, ICAM-1, PPAR-*γ*, and *β*-actin (Santa Cruz Biotechnology, Santa Cruz, CA) at a final dilution of 1 : 1000 overnight at 4°C. The blot was washed several times with PBS-T and incubated with the appropriate horseradish peroxidase-conjugated secondary antibody for 1 h. After the membrane was washed several times with PBST, the bound secondary antibody was detected by enhanced chemiluminescence (Amersham, Buckinghamshire, UK). Protein expression levels were determined by analyzing the signals captured on the nitrocellulose membrane using a ChemiDoc image analyzer (Bio-Rad).

### 2.11. Statistical Analysis

Values are shown as mean ± S.E. Statistical analyses were performed using analysis of variance followed by Student's *t*-test and one-way ANOVA. Differences with a value of *P* < 0.05 were considered statistically significant.

## 3. Results

### 3.1. Effect of DYSGT on Changes in Body Weight, Systolic Blood Pressure, and Plasma Biomarker Levels

At time of sacrifice, mean body weight was as shown in Table 2, WTD-fed ApoE KO mice showed significantly increased body weight compared with RD-fed control mice. However, there were no differences of body weight among the WTD-fed ApoE KO mice groups. The systolic blood pressure (SBP) level after 12 weeks was significantly decreased in DYSGT-treated ApoE KO mice compared with the untreated WTD-fed ApoE KO mice (*P* < 0.01). As similar in level, rosiglitazone-treated ApoE KO mice indicated a remarkable decrease in that level. 

The BUN, LDL cholesterol, and TG levels of blood plasma were significantly increased in WTD-fed ApoE KO mice compared with RD-fed control mice. However, biochemical analysis of blood samples of ApoE KO mice showed that administration of DYSGT at a dose of 200 mg/kg/day resulted in a significant decrease of BUN (1.32 ± 0.05 versus 0.7 ± 0.04 mg/dL, *P* < 0.05) in comparison with ApoE KO (nontreated) mice. Moreover the administration of DYSGT resulted in a significant decrease of LDL-cholesterol (1.02 ± 0.06 versus 0.86 ± 0.03 *μ*g/*μ*L, *P* < 0.05), and TG levels (102.42 ± 6.67 versus 47.83 ± 5.55 mg/dL, *P* < 0.01) when compared with ApoE KO mice. However, HDL-cholesterol level was not increased in DYSGT. Similarly, in Ros-treated ApoE KO mice, BUN, LDL cholesterol, and TG levels were significantly lower than those levels of ApoE KO mice.

### 3.2. Effect of DYSGT on Blood Glucose and Glucose Tolerance Test Levels

**Figure 1:**
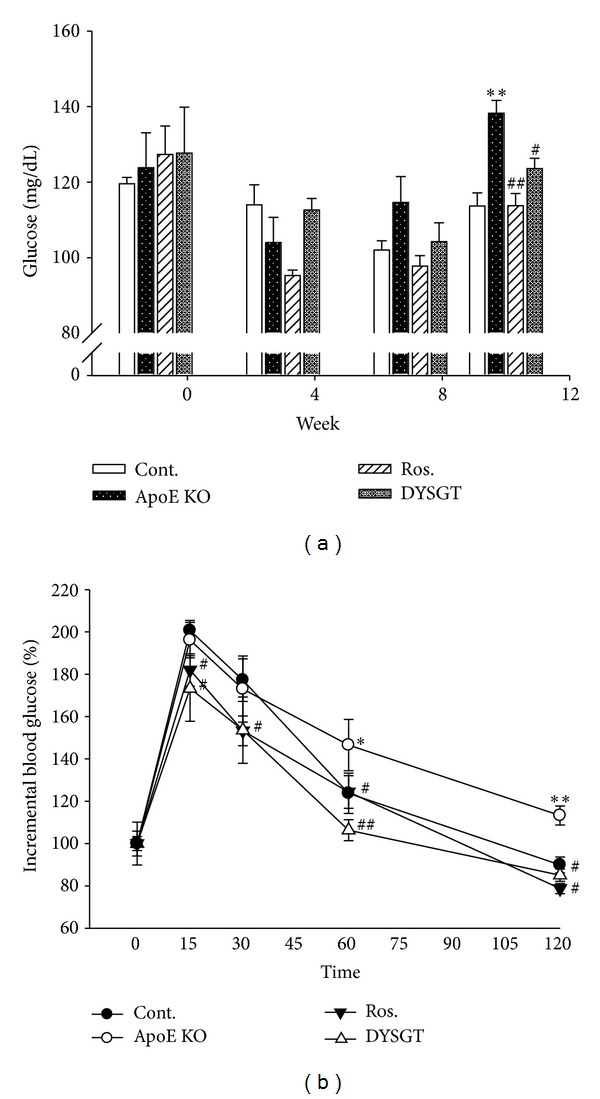
Effect of DYSGT on blood glucose levels (a) and glucose tolerance test (b) in ApoE KO mice. Values are expressed as mean ± SE values (*n* = 12); *P < 0.05, **P < 0.01 versus cont.; ^#^ *P* < 0.05, ^##^ *P* < 0.01 versus ApoE KO.

Fasted blood glucose level of untreated WTD-fed ApoE mice was significantly higher than that of the RD-control mice at the 12 weeks. Interestingly, the blood glucose levels were markedly reduced in DYSGT-treated ApoE KO mice (*P* < 0.05) (Figure 1(a)). Glucose tolerance was significantly better in DYSGT-treated ApoE KO mice than in untreated WTD-fed ApoE mice, as shown by the much smaller rise in blood glucose in DYSGT-treated ApoE KO mice over the investigated 15 min time period following administration of 1 g/kg glucose (Figure 1(b)). Similarly, these levels of Ros-treated ApoE KO mice were significantly decreased compared with untreated WTD-fed ApoE mice.

### 3.3. Reduction in Lipid Accumulation in the Aorta and Aortic Valve

**Figure 2:**
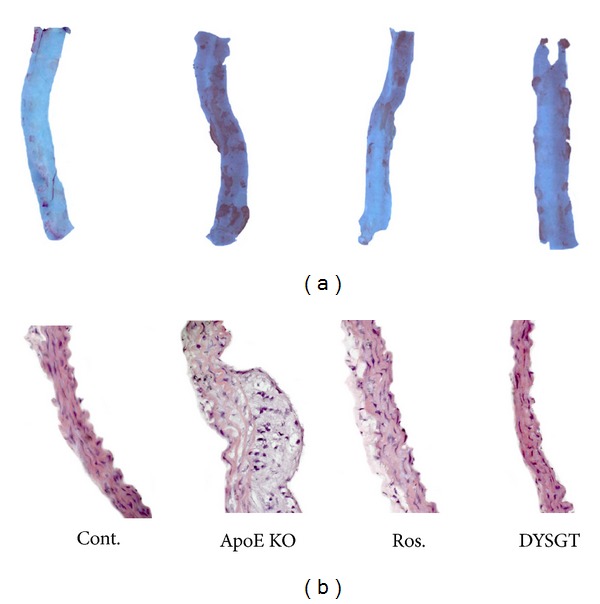
Atherosclerotic lesions in aortic root and aorta of ApoE KO mice. The aorta was obtained from mice fed a regular diet or western diet with or without oral administration of DYSGT (200 mg/kg/day) for 12 weeks (*n* = 6–8). Representative photomicrographs of oil red O (pink color) staining (a) and H&E staining (b) in cross-sections of descending aorta (100x magnification).

To investigate whether DYSGT treatment could inhibit lipid accumulation in the aorta of ApoE KO mice fed a western diet, we assessed Oil Red O staining. Consistent with the change in lipid profile, western diet in ApoE KO mice induced lipid-rich plaque, whereas treatment with DYSGT significantly inhibited the development of atherosclerosis (Figure 2(a)). Microscopic examination of H&E staining revealed that atherosclerotic lesions such as roughened endothelial layers and fibrous cap formation were shown in WTD-fed ApoE KO mice. However, chronic treatment with DYSGT maintained the smooth and soft character of the tunica intima and decreased the intima-media thickness in aortic sections (Figure 2(b)). 

### 3.4. Effect of DYSGT on Changes in Vascular Tone of WTD-Fed ApoE KO Mice

**Figure 3:**
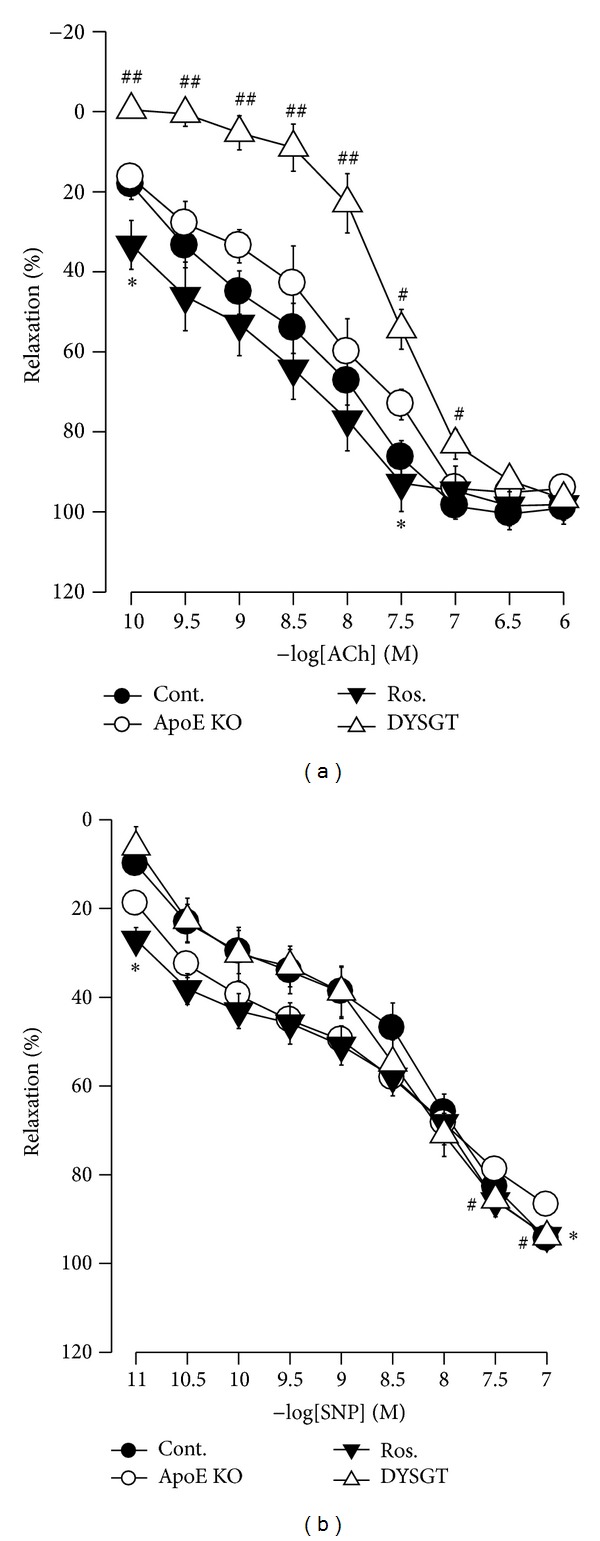
Effects of DYSGT on relaxation of thoracic aorta induced by (a) acetylcholine or (b) sodium nitroprusside (SNP) in WTD-fed ApoE KO mice. Data are mean ± S.E. values (*n* = 5). *P < 0.05, versus cont.; ^#^ *P* < 0.05, versus ApoE KO.

 Vasorelaxation responses to ACh were measured in the thoracic aorta of WTD-fed ApoE KO mice (Figure 3(a)). Significant impairment of vasorelaxation was evident in thoracic aorta of WTD-fed ApoE KO mice compared with RD-fed control mice. DYSGT treatment restored the vasorelaxation response. On the other hand, the vasorelaxant response to sodium nitroprusside (SNP), a NO donor, was unchanged, and DYSGT and rosiglitazone did not affect this response (Figure 3(b)). 

### 3.5. Effect of DYSGT on Vascular Inflammation in Aortic Tissue

**Figure 4:**
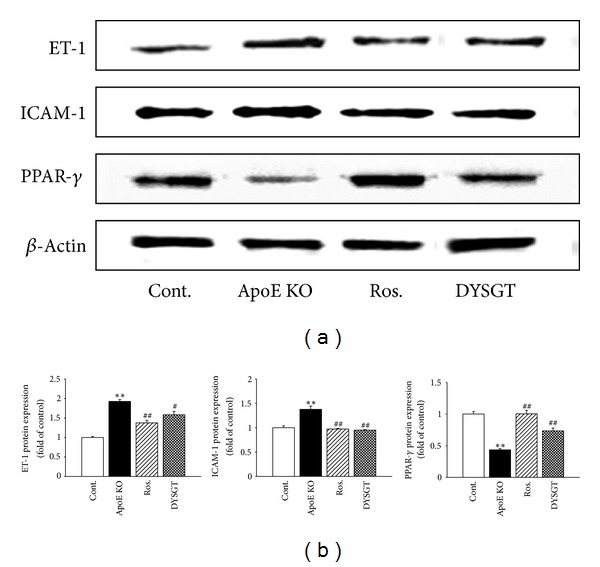
Effect of DYSGT on ET-1, ICAM-1, and PPAR-*γ* protein expression in the aorta of ApoE KO mice. Western blots and corresponding densitometric analyses of ET-1, ICAM-1, and PPAR-*γ* in aortic tissue. Values are expressed as mean ± S.E. (*n* = 4); **P < 0.01 versus cont.; ^#^ *P* < 0.05, ^##^ *P* < 0.01 versus ApoE KO.

The protein expression of ET-1, ICAM-1, and PPAR-*γ* in the descending aortas of all groups of mice was examined by Western blot analysis. Expression of ET-1, ICAM-1 in WTD-fed ApoE KO mice was significantly increased compared with control mice fed RD. The group treated with DYSGT at 200 mg/kg/day showed a lower band intensity of ET-1 and ICAM-1 compared with untreated WTD-fed ApoE KO mice. Densitometric analysis indicated that DYSGT significantly decreased ET-1 (82.09%) and ICAM-1 (69.02%) protein expression compared with non-treated ApoE KO mice (100%) (Figure 4(a)). Conversely, the PPAR-*γ* protein expression was suppressed in the aorta of WTD-fed compared with RD-fed control mice. Rosiglitazone and DYSGT treatment markedly restored PPAR-*γ* expression levels by 1.0-fold and 0.73-fold (WTD-fed ApoE KO = 0.4-fold), respectively (*P* < 0.01) (Figure 4(b)).

**Figure 5:**
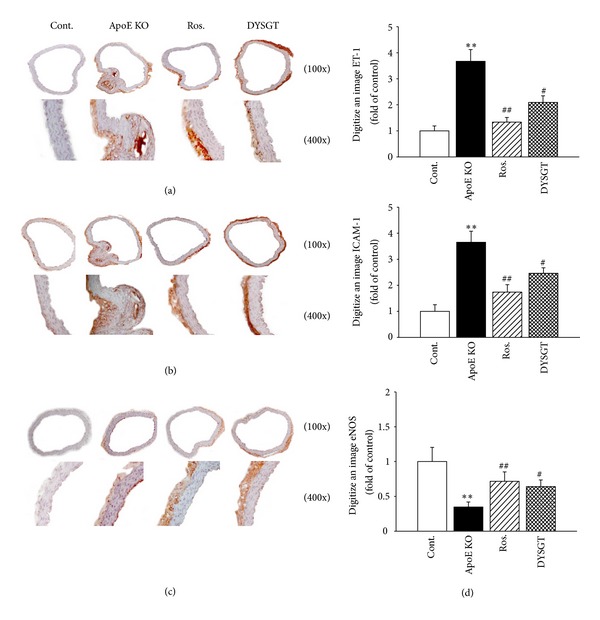
Effect of DYSGT on (a) ET-1, (b) ICMA-1, and (c) eNOS immunoreactivity in aorta of ApoE KO mice. Immunohistochemical staining of ET-1, ICMA-1, and eNOS in aorta from cont., ApoE KO mice, ApoE mice treated with rosiglitazone, and ApoE mice treated with DYSGT. (d) Quantitative analysis of ET-1, ICMA-1, and eNOS positive area, respectively. The average score of 5–8 randomly selected sites per section of aorta was calculated. Data expressed as mean ± S.E.; **P < 0.01 versus cont.; ^#^ *P* < 0.05, ^##^ *P* < 0.01 versus ApoE KO. Original magnification, 100x and 400x.

**Figure 6:**
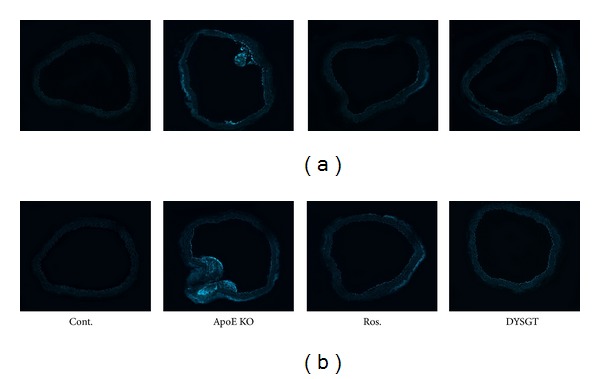
Immunofluorescence staining of (a) ICAM-1 and (b) ET-1 in the aorta. Representative histological sections are thoracic aorta of Cont., ApoE KO mice, ApoE mice treated with rosiglitazone, and ApoE mice treated with DYSGT incubated with anti-ICAM-1, anti-ET-1 antibodies, respectively. Original magnification: 100x.

Figure 5 shows representative micrographs of ET-1, ICAM-1, and eNOS expression using immunohistochemistry. As a result, in the control group, expression of ET-1 was observed in blood vessel intima (atherosclerotic lesion) and in endothelium covering atherosclerotic lesion as well as in endothelium outside the lesion. DYSGT treatment markedly decreased the levels of ET-1 by 57%, respectively (Figures 5(a) and 5(d)). Similarly, ICAM-1 was significantly increased in WTD-fed ApoE KO mice. Treatment with rosiglitazone and DYSGT significantly decreased ICAM-1 expression by 48% and 67%. (Figures 5(b) and 5(d)). The eNOS expression was suppressed in the aorta of WTD-fed ApoE KO mice compared with RD-fed control mice (Figure 5(c)). DYSGT treatment markedly restored eNOS expression levels by 1.9-fold, respectively (*P* < 0.05) (Figure 5(d)). Similarly, in WTD-fed ApoE KO mice, immunofluorescence of the aorta tissues showed that ICAM-1 and ET-1 expression were decreased in rosiglitazone and DYSGT groups compared with ApoE KO group (Figure 6).

## 4. Discussion

In the present study, we investigated the protective role of DYSGT in diabetic atherosclerosis using western diet-ApoE KO mice. In addition to the prevention of atherosclerosis, we clearly demonstrated improvement of endothelial dysfunction. Atherosclerosis is a consequence of chronic inflammation of the vessel wall [3]. One of the key events is the recruitment of leukocytes and the adhesion of platelets to the endothelium overlying the plaque [17–19]. The present study demonstrated that DYSGT significantly reduced blood glucose levels in WTD-fed ApoE KO mice. Glucose tolerance also was significantly better in DYSGT, suggesting a beneficial effect of insulin resistance. Thus, further study is required to clarify the activation of insulin signaling induced by DYSGT. Following administration of DYSGT for 12 weeks, there was no difference of body weight. However, it is clear that DYSGT markedly reduced LDL cholesterol, triglyceride levels. Consistent with increasing LDL cholesterol and triglyceride, lipid accumulation in the thoracic aorta was shown in WTD-fed ApoE mice. We suspected that western diet/hyperlipidemia-induced obesity is dependent of various administration periods (e.g., >12 weeks). At least, it is clear that DYSGT attenuated lipid accumulation and atherosclerotic lesions in the thoracic aorta. These results suggest that DYSGT is specific to vessel leading to antiatherosclerosis property. 

The vascular endothelium, which lies between circulating blood and vascular smooth muscle and senses changes or abnormalities in blood flow and blood pressure, plays an important role in modulation of vascular tone [20–22]. In our results, blood pressure was determined using the tail-cuff technique. The mean SBP with 12 weeks of WTD-fed ApoE KO mice was significantly increased; however, DYSGT significantly decreased this trend. In addition, WTD-fed ApoE KO mice also caused endothelial dysfunction as evidenced by decreased ACh-induced vascular tone and increased ET-1 expression. DYSGT exerted endothelium-dependent vasodilation in thoracic aortic smooth muscle. There were no significant differences of sodium SNP-induced dilation between DYSGT treated groups and the control group. These findings suggest that the hypotensive effects of DYSGT are mediated by ACh and further via the endothelium-dependent NO/cGMP pathway. In fact, other studies have also reported defective acetylcholine response without a corresponding change in SNP response in aortas of obese rats fed a high fat diet, and impaired relaxation of the aorta induced by acetylcholine but not SNP has been seen in obese Zucker rats as a consequence of endothelial dysfunction [23, 24]. It has been well documented that endothelium-dependent vascular relaxation is abnormal in both hypercholesterolemia and atherosclerosis because the ability of NO to maintain vascular tone is impaired [25, 26]. In addition, DYSGT reduced blood pressure via the inhibition of ET-1 expression. Thus, we suggest a protective role of DYSGT on vasoconstriction-mediated hypertension, and further progression to vascular dysfunction in diabetic atherosclerosis model.

Endothelial dysfunction will include not only reduced vasodilation but also vascular inflammation and atherosclerotic lesions [27, 28]. Blocking of inflammatory mediators can decrease the size of the atherosclerotic lesion. Adhesion molecules such as VCAM-1 and ICAM-1 play a significant role in the process of atherosclerosis as they ensure the recruitment of inflammatory cells. The study of ICAM-1 expression was reported by several authors in various models of atherosclerosis [29–31]. It has been shown that ICAM-1 is detected in the regions predisposed to atherosclerotic lesion formation in normocholesterolemic rabbits, and the expression of both molecules is upregulated by a high-cholesterol diet in rabbits and mice [29]. To examine the effect of DYSGT on vascular inflammation, adhesion molecule ICAM-1 expression was measured in the thoracic aorta. The WTD-fed ApoE KO mice had significantly increased levels of aortic expression of ICAM-1. However, this increase was significantly reduced by DYSGT. These data suggested that vascular inflammation is related to vasoconstriction; that is, eNOS-mediated NO production is required to defend against diabetic atherosclerosis, especially vascular dysfunction. We previously reported that *Prunella vulgaris* exerts anti-inflammatory effect by inducing eNOS expression in vascular endothelial cell [32]. Furthermore, *Prunella vulgaris*-induced eNOS/NO expression is involved in repairment of vasodilation and vascular inflammation in db/db mice [33]. In fact, NO, an important physiological regulator of vascular homeostasis, is implicated in the pathophysiology of atherosclerosis [6, 7]. Recent study shows that both eNOS and nNOS significantly inhibit atherosclerosis in ApoE KO mice [34, 35]. Thus, we could not rule out the involvement of nNOS in the improvement of present animal model. Next study will be clarify the possible role of DYSGT in the activation of specific NOS for the involvement of vascular protection against diabetic atherosclerosis. Another defense system is PPAR-*γ*, potent antidiabetic drug, since both eNOS and PPAR-*γ* expression were recovered by DYSGT. Effect of DYSGT in type II diabetes is similar to rosiglitazone which is ameliorated in insulin sensitivity. However, recently reported side effect, weight gain or heart attack, is different although the present study did not show those effects [11, 12]. Thus, these finding suggested that DYSGT is a safe and potent traditional drug for the treatment of vascular dysfunction in diabetic atherosclerosis. 

Doinseunggitang (DYSGT), traditional prescription, has been used for promoting blood circulation to remove blood stasis. In our study, treatment with DYSGT in WTD-fed ApoE KO mice reduced hypertension as well as insulin resistance. DYSGT also improved LDL cholesterol and triglyceride levels and reduced vascular inflammation. Thus, to our knowledge, this study provides the first evidence that Doinseunggitang apparent antihypertensive, hyperlipidemic, and antivascular inflammatory effects are in agreement with traditional medicinal effect. Therefore, these findings, at least in part, indicate that Doinseunggitang protects vascular function against initiation and development of atherosclerosis.

## Figures and Tables

**Table 1:** Anhydrous milk fat typically contains approximately 0.3% cholesterol. On this basis, D12079B contains approximately 0.21% cholesterol.

		gm %	kcal %
Protein		20	17
Carbohydrate		50	43
Fat		21	41
	Total kcal/gm	4.7	100

Ingredient		gm	kcal

Casein, 80 Mesh		195	780
DL-Methionine		3	12
Corn starch		50	200
Maltodextrin 10		100	400
Sucrose		341	1364
Cellulose		50	0
Milk fat, anhydrous*		200	1800
Corn oil		10	90
Mineral Mix S10001		35	0
Calcium carbonate		4	0
Vitamin Mix V10001		10	40
Choline bitartrate		2	0
Cholesterol, USP*		1.5	0
Ethoxyquin		0.04	0

Total		1001.54	4686

**Table 2:** Effect of DYSGT on body weight, blood pressure, and plasma biomarker levels in ApoE KO mice.

	Parameter						
	Body weight (g)		Systolic blood pressure (mmHg)		BUN	LDL cholesterol	TG
	Start	Final	Start	Final	(mg/dL)	(*μ*g/*μ*L)	(mg/dL)
Cont.	23.09 ± 0.90	28.58 ± 0.98	103.92 ± 2.00	105.42 ± 1.80	0.67 ± 0.05	0.19 ± 0.02	44.92 ± 4.81
ApoE KO	22.76 ± 0.83	37.18 ± 0.88**	103.08 ± 2.69	118.92 ± 2.26**	1.32 ± 0.05*	1.02 ± 0.06**	102.42 ± 6.67**
Ros.	21.96 ± 0.44	35.3 ± 0.87	100.92 ± 2.04	105.58 ± 1.69^##^	0.60 ± 0.03^#^	0.84 ± 0.04^#^	51.75 ± 5.38^##^
DYSGT	21.92 ± 0.66	35.17 ± 1.32	100.33 ± 3.35	106.25 ± 2.52^##^	0.70 ± 0.04^#^	0.86 ± 0.03^#^	47.83 ± 5.55^##^

Data are mean ± SE values (*n* = 12); *P < 0.05, **P < 0.01 versus control; ^#^ *P* < 0.05, ^##^ *P* < 0.01 versus ApoE KO. Cont.: control; ApoE KO: apolipoprotein E knockout; Ros: rosiglitazone; DYSGT: Doinseunggitang.
